# Venous thromboembolism with modern glucose-lowering agents in diabetes: active-comparator evidence beyond placebo-based meta-analyses

**DOI:** 10.1007/s11739-026-04363-5

**Published:** 2026-05-06

**Authors:** Andrea Boccatonda, Chiara Simion, Alice Brighenti, Damiano D’Ardes, Fabio Piscaglia, Carla Serra, Paolo Simioni, Elena Campello

**Affiliations:** 1 Diagnostic and Therapeutic Interventional Ultrasound Unit, IRCCS Azienda Ospedaliero-Universitaria Di Bologna, Policlinico Sant’Orsola-Malpighi, Via Massarenti N 9, 40138 Bologna, Italy; 2 Internal Medicine, Thrombotic and Hemorrhagic Diseases Unit, Department of Medicine, Padova University Hospital, Padua, Italy; 3 Institute of “Clinica Medica”, Department of Medicine and Aging Science, G. D’Annunzio University of Chieti-Pescara, Chieti, Italy; 4 Division of Internal Medicine, Hepatobiliary and Immunoallergic Diseases, IRCCS Azienda Ospedaliero-Universitaria Di Bologna, Bologna, Italy; 5 Department of Medical and Surgical Sciences, University of Bologna, Bologna, Italy

**Keywords:** VTE, DVT, PE, Diabetes, GLP1-RA, SGLT2—1

## Abstract

The influence of newer glucose-lowering agents on venous thromboembolism (VTE) risk has important therapeutic implications. This meta-analysis compared sodium-glucose cotransporter-2 inhibitors (SGLT2—i) and glucagon-like peptide-1 receptor agonists (GLP1-RA) with dipeptidyl peptidase-4 inhibitors (DPP-4i) and evaluated head-to-head SGLT2i versus GLP1-RA comparisons. Eligible studies enrolled adults with diabetes and reported adjusted relative risks for VTE, pulmonary embolism (PE), or deep vein thrombosis (DVT) using new-user cohort, target-trial emulation, nested case–control, or randomized controlled designs. Pooled estimates were derived using inverse-variance meta-analysis on the log scale, with random-effects sensitivity analyses. Six studies met inclusion criteria, all of moderate risk of bias. Compared with DPP-4i, both SGLT2—i (HR 0.72; 95% CI 0.62–0.82) and GLP1-RA (HR 0.78; 95% CI 0.73–0.83) were associated with significantly reduced VTE risk, whereas no difference was observed between SGLT2—i and GLP1-RA (HR 1.02; 95% CI 0.84–1.23). Component analyses were directionally consistent: for PE, SGLT2—i versus DPP-4i HR 0.44 (95% CI 0.30–0.64) and GLP1-RA versus DPP-4i HR 0.74 (95% CI 0.68–0.82); for DVT, SGLT2—i versus DPP-4i HR 0.82 (95% CI 0.68–0.99) and GLP1-RA versus DPP-4i HR 0.81 (95% CI 0.75–0.88). In adults with diabetes, SGLT2—i and GLP1-RA were each associated with a lower risk of VTE compared with DPP-4i, while head-to-head comparisons showed no clear difference and were limited by imprecision. These findings should be interpreted with caution given the limited evidence base. Overall, they suggest that concerns regarding VTE risk should not be a primary driver of treatment selection between these agents in clinical practice.

*Trial registration:*PROSPERO CRD4201149607.

**Graphical abstract:**

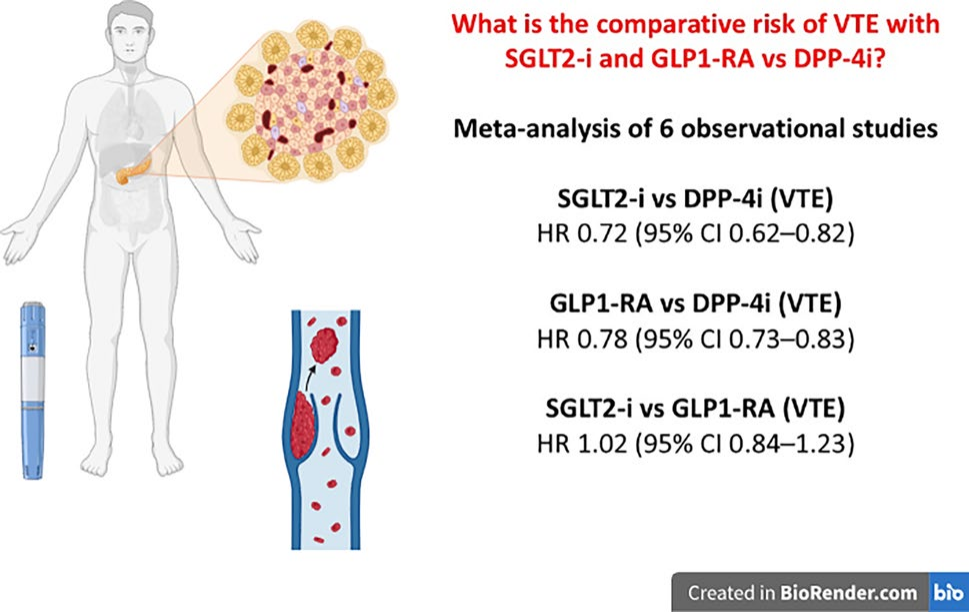

In a meta-analysis of six observational studies, both SGLT2 inhibitors and GLP-1 receptor agonists were associated with a lower risk of venous thromboembolism compared with DPP-4 inhibitors, while no significant difference was observed between SGLT2 inhibitors and GLP-1 receptor agonists in head-to-head comparisons.

**Supplementary Information:**

The online version contains supplementary material available at 10.1007/s11739-026-04363-5.

## Introduction

Glucagon-like peptide-1 receptor agonists (GLP1-RAs) and sodium-glucose cotransporter 2 inhibitors (SGLT2—i) are cornerstone therapies for type 2 diabetes, with established cardiovascular and renal benefits. Less clear is their risk profile for venous thromboembolism (VTE) (deep vein thrombosis [DVT] and pulmonary embolism [PE]), a clinically relevant outcome given the increased thrombotic risk associated with diabetes, obesity, and related comorbidities [1–6]. The available comparative evidence is fragmented: it includes observational head-to-head studies and comparisons with active comparators, primarily DPP-4 inhibitors (DPP-4i) [7–9] as well as specific contexts such as the orthopedic perioperative setting [10].

From a biological perspective, several mechanisms have been hypothesized through which modern glucose-lowering agents could influence VTE risk. Type 2 diabetes is characterized by a prothrombotic milieu, including endothelial dysfunction, low-grade inflammation, platelet hyperreactivity, and impaired fibrinolysis, which may contribute to venous thrombogenesis [11–13]. GLP1-RAs have been shown to exert anti-inflammatory and vasculoprotective effects, improve endothelial function, and reduce markers of oxidative stress, which could theoretically mitigate thrombotic risk [14, 15]. In addition, weight loss and improvements in insulin resistance associated with GLP1-RAs may indirectly reduce VTE risk by attenuating obesity-related hypercoagulability [14, 15]. SGLT2—i, on the other hand, induce hemoconcentration through osmotic diuresis and increase hematocrit, raising theoretical concerns regarding thrombosis [16–18]. However, they also improve endothelial function, reduce systemic inflammation, and may favorably modulate fibrinolytic balance [16, 17]. Observational data suggest that SGLT2—i–associated erythrocytosis is not accompanied by an increased risk of venous or arterial thrombosis, indicating that these competing mechanisms may offset each other in clinical practice [18].

Recently, two meta-analyses of randomized controlled trials (RCTs) have been published addressing the association between GLP1-RAs and VTE, with partially divergent findings depending on the outcome component and choice of comparator [19, 20]. The first pooled 27 RCTs (≈84,000 participants) and found no significant difference in overall VTE risk with GLP-1RAs compared with placebo, while identifying a reduction in pulmonary embolism (PE) (RR ≈0.60; 95% CI 0.39–0.94) [19]. The second included 39 RCTs (≈70,500 participants) with “placebo or non–GLP-1RA drugs” as comparators and reported a non-significant trend toward higher overall VTE risk but a significant increase in DVT (OR 1.64; 95% CI 1.14–2.36), especially in longer-term trials (> 1.5 years) and cardiovascular outcome trials (CVOTs) [20]. These trial-based findings coexist with large-scale observational evidence, such as a target trial emulation including over 270,000 matched patients, which demonstrated a consistent reduction in VTE risk with GLP-1RAs compared to DPP-4i (HR 0.78; 95% CI 0.73–0.83) [14].

The main aim of our work was to systematically assess and quantify the risk of VTE associated with GLP1-RA and SGLT2—i compared with active comparators (DPP-4i and between each other), also considering individual components (PE, DVT). This review was prospectively registered in PROSPERO: CRD4201149607.

## Methods

### Eligibility criteria

We searched the literature from database inception to September 16, 2025, for articles in English, limiting inclusion to comparative human studies (age ≥ 18 years). Both observational studies (new-user cohorts, nested case–control, target trial emulations) and any RCTs reporting VTE outcomes were considered.

### Inclusion criteria

Studies were considered eligible if they included adult participants (≥ 18 years) receiving antidiabetic therapy or exposed to GLP1-RA or SGLT2—i in any clinical setting, including perioperative care. Eligible studies specifically evaluated exposure to GLP1-RA and/or SGLT2-I and reported outcomes relevant to VTE. To ensure appropriate comparisons, only studies including an active comparator were considered, namely DPP-4i or head-to-head comparisons between GLP1-RA and SGLT2—i. Both randomized and non-randomized study designs were eligible. In particular, we included randomized controlled trials (RCTs) reporting VTE outcomes, as well as observational studies such as new-user cohort studies, nested case–control studies, and target trial emulations.

### Exclusion criteria

We excluded non-human studies, participants aged < 18 years, and studies conducted exclusively in pregnancy or pediatric populations. Studies in which exposure started after a VTE event (reverse causation) were also excluded. We further excluded studies without exposure to GLP1-RA or SGLT2—i, as well as studies reporting mixed drug classes only as aggregates (e.g., “incretin-based therapies”) without extractable class-specific results. Studies without an eligible active comparator (DPP-4i or head-to-head GLP1-RA versus SGLT2—i comparisons) were excluded, as were placebo-only trials unless they allowed a direct active-versus-active estimate. We also excluded studies that did not report VTE outcomes (composite VTE, PE, or DVT), reported only surrogate or biomarker outcomes, combined venous and arterial events without stratification, or did not provide effect estimates with insufficient data to compute adjusted HRs, RRs, or ORs. Finally, we excluded non-comparative designs (including case reports, case series, and single-arm studies), cross-sectional and ecological studies, disproportionality analyses from spontaneous reporting systems without a comparative design, as well as narrative reviews, editorials, guidelines, protocols, and conference abstracts lacking sufficient extractable data.

### Information sources

MEDLINE/PubMed, Embase, Web of Science Core Collection, Scopus, Cochrane Library. Grey literature/preprints: medRxiv and Research Square (title/abstract screening).

### Search strategy

The strategies were adapted to each database using controlled vocabularies (MeSH/Emtree) and free-text terms. A detailed search strategy is available in the Supplementary Material.

### Selection process and data collection process

Study selection was performed in two stages. First, titles and abstracts were independently screened by two reviewers (A.B. and C.S.) to identify potentially eligible studies. Full texts of all records deemed relevant or uncertain were then independently assessed by the same two reviewers against the predefined inclusion and exclusion criteria. Discrepancies at either stage were resolved through discussion, and when consensus could not be reached, a third senior reviewer (E.C.) was consulted.

### Data items

We extracted study-level and comparison-level data in duplicate, including study design, population characteristics, exposure and comparator definitions, follow-up, and outcome definitions. Adjusted effect estimates (preferentially HRs) with 95% confidence intervals were collected, prioritizing the most fully adjusted models and primary analysis definitions. Additional details on extraction rules and prioritization criteria are provided in the Supplementary Material.

### Study risk of bias assessment

We assessed the risk of bias in non-randomized studies using the ROBINS-I tool, evaluating all seven standard domains. Two reviewers independently performed the assessments, with disagreements resolved through discussion or consultation with a third reviewer. The overall risk of bias for each study was determined based on the domain judged to be at the highest risk. Given that both randomized and non-randomized studies were considered eligible a priori, we planned to assess the risk of bias in RCTs using the Cochrane Risk of Bias 2 (RoB 2) tool.

### Effect measures and synthesis methods

The primary effect measure was the adjusted HR. When studies reported adjusted relative risks (RRs) or odds ratios (ORs), these were synthesized on the log scale using a generic inverse-variance approach; given the low incidence of VTE, ORs were considered reasonable approximations of RRs. For each study, we preferentially extracted the most fully adjusted estimate and the longest available follow-up. Adjusted log-effect estimates were pooled using inverse-variance fixed-effect models as the primary approach, while random-effects models were prespecified as sensitivity analyses. Statistical heterogeneity was assessed using Cochran’s Q, I2, and τ2, and, where at least two studies were available, 95% prediction intervals were calculated.

Analyses were conducted at the class level. Sensitivity analyses were performed restricting to studies that adjusted for body mass index (BMI) or adiposity. In addition, we explored the impact of follow-up duration on effect estimates. For each study, we extracted the mean or median follow-up (or, when applicable, the design-imposed time horizon, such as in target trial emulations), and conducted subgroup analyses by follow-up length (≤ 12 months vs > 12 months or variable), as well as random-effects meta-regression using follow-up duration (in months) as a continuous moderator.

### Reporting bias assessment

Small-study effects were assessed with funnel plots and Egger’s test, where k ≥ 3; tests are underpowered at *k* = 3.

### Certainty assessment

We appraised the certainty of evidence using the GRADE framework at the contrast-outcome level (e.g., SGLT2—i vs DPP-4i for VTE, PE, DVT). Because all included studies are observational, certainty starts at low and can be rated down for: (1) risk of bias (ROBINS-I), (2) inconsistency, (3) indirectness, (4) imprecision, and (5) publication bias; and up for large effects, dose–response, or when all plausible confounding would reduce an observed benefit.

## Results

### Study selection

**Fig. 1:**
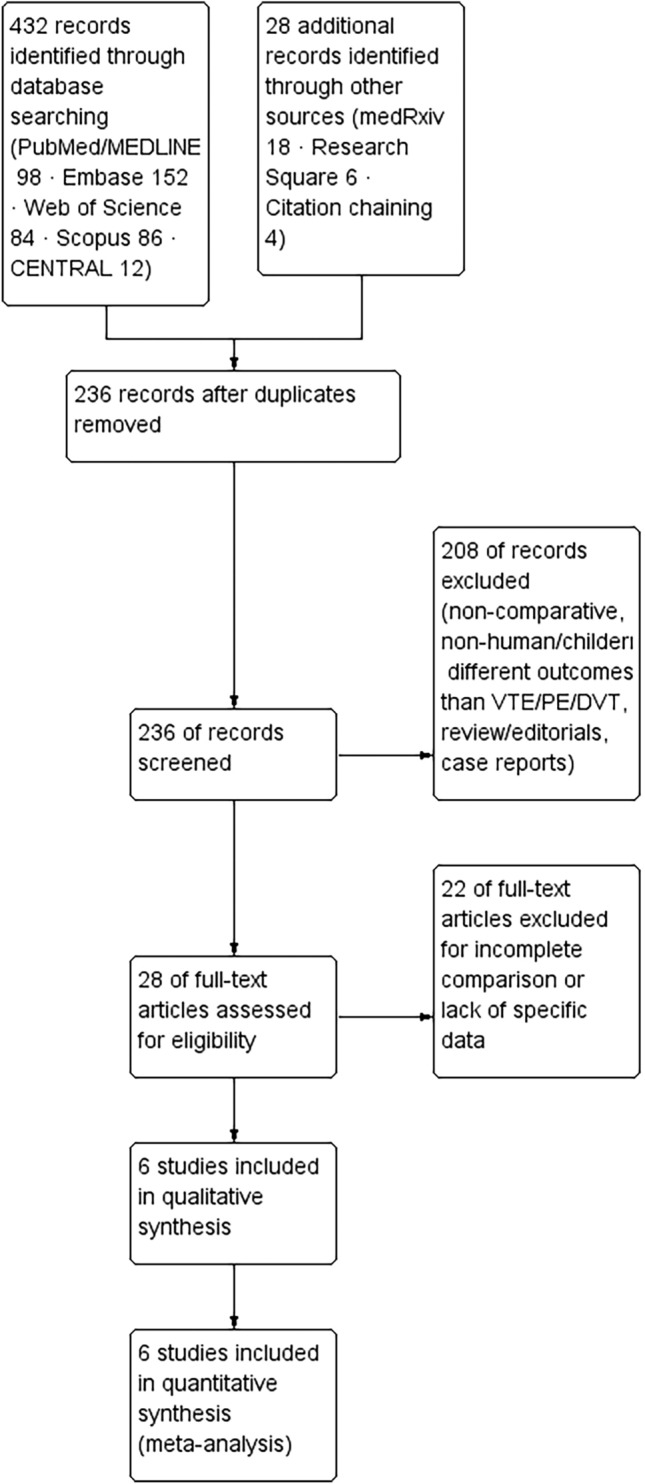
Study flow diagram

The search retrieved 432 records from bibliographic databases (PubMed/MEDLINE 98, Embase 152, Web of Science 84, Scopus 86, Cochrane CENTRAL 12) and 28 additional records from other sources. After de-duplication, 236 records remained and were screened by title/abstract; 208 were excluded as non-comparative, non-human/children, reporting outcomes other than VTE/PE/DVT, or being reviews/editorials/case reports. We assessed 28 full texts for eligibility, of which 22 were excluded due to incomplete comparator structures or insufficient outcome data. Six studies met the inclusion criteria for the qualitative synthesis, and all six contributed data to the quantitative synthesis (meta-analysis) [14, 21–25]. The selection process is summarized in Fig. 1.

### Study characteristics

Six comparative studies met the inclusion criteria, spanning 2018–2025 and covering Europe, North America, and Asia (Table 1). All were observational and used active comparators; five were cohort-based (new-user, PS-matched/weighted or target-trial emulations) and one was a nested case–control within a new-user cohort. Follow-up ranged from roughly 9 to 12 months in the primary analyses.

**Table 1:** Summary of findings of studies included in the meta-analysis

Study	Comparison	Outcome	EFFECT_TYPE	Effect	CI_LOW	CI_HIGH	Patients	Design	COUNTRY_DB	FOLLOW_UP_MO	Notes
**Schmedt 2021 **[23]	SGLT2—i vs DPP-4i	VTE	RR	0.75	0.59	0.94	Source cohort: 219,538 people with T2D; VTE cases: 2,152; controls: 85,104	Nested case–control in new-user cohort	Germany, InGef claims	Not fixed	Risk-set sampling; current-use exposure windows
**Aloe 2023 **[21]	SGLT2—i vs DPP-4i	VTE	HR	0.65	0.34	1.25	5414 SGLT2—i users vs 5414 DPP-4i users	Prevalent/incident new-user, active-comparator, HDPS	UK, CPRD + HES/ONS	10.0	Matched & HDPS adjusted
**Chiang 2025 **[14]	GLP-1 RA vs DPP-4i	VTE	HR	0.78	0.73	0.83	270,129 GLP1-RA users vs 270,129 DPP-4i users	Target Trial Emulation, PS-matched	USA (multi-EHR)	12.0	Primary
**Chiang 2025 **[14]	GLP-1 RA vs DPP-4i	PE	HR	0.74	0.68	0.82	270,129 GLP1-RA users vs 270,129 DPP-4i users	Target Trial Emulation, PS-matched	USA (multi-EHR)	12.0	Component
**Chiang 2025 **[14]	GLP-1 RA vs DPP-4i	DVT	HR	0.81	0.75	0.88	270,129 GLP1-RA users vs 270,129 DPP-4i users	Target Trial Emulation, PS-matched	USA (multi-EHR)	12.0	Component
**Patil 2023 **[22]	SGLT2—i vs GLP-1 RA	VTE	HR	1.02	0.80	1.30	35,347 SGLT2—i users vs 35,347 GLP1-RA users	New-user, PS-matched (active comparator)	USA, Veterans Health Administration (VHA)	~ 12.1 (SGLT2—i) / ~ 18.5 (GLP-1 RA)	Predominantly male cohort; outcome based on ICD codes
**Tsai 2023 **[24]	SGLT2—i vs DPP-4i	VTE	HR	0.70	0.59	0.84	IPTW weighted pseudo-populations: 134,215 SGLT2—i users vs 598,904 DPP-4i users	New-user, stabilized IPTW	Taiwan NHIRD	Variable	Weighted pseudo-populations; ICD-9/10 outcomes
**Tsai 2023 **[24]	SGLT2—i vs DPP-4i	PE	HR	0.44	0.30	0.64	IPTW weighted pseudo-populations: 134,215 SGLT2—i users vs 598,904 DPP-4i users	New-user, stabilized IPTW	Taiwan NHIRD	Variable	Weighted pseudo-populations; ICD-9/10 outcomes
**Tsai 2023 **[24]	SGLT2—i vs DPP-4i	DVT	HR	0.82	0.68	0.99	IPTW weighted pseudo-populations: 134,215 SGLT2—i users vs 598,904 DPP-4i users	New-user, stabilized IPTW	Taiwan NHIRD	Variable	Weighted pseudo-populations; ICD-9/10 outcomes
**Tsai 2023 **[24]	SGLT2—i vs GLP-1 RA	VTE	HR	1.39	0.32	5.94	136,612 SGLT2—i users vs 5339 GLP1-RA users	New-user (head-to-head, small GLP-1 RA cohort)	Taiwan NHIRD	Variable	Events 178 vs 4; PY 176,225 vs 5,601
**Tsai 2023 **[24]	SGLT2—i vs GLP-1 RA	DVT	HR	3.48	0.49	24.91	136,612 SGLT2—i users vs 5339 GLP1-RA users	New-user (head-to-head, small GLP-1 RA cohort)	Taiwan NHIRD	Variable	Events 155 vs 2; PY 176,241 vs 5,601
**Tsai 2023 **[24]	SGLT2—i vs GLP-1 RA	PE	HR	0.42	0.06	3.06	136,612 SGLT2—i users vs 5339 GLP1-RA users	New-user (head-to-head, small GLP-1 RA cohort)	Taiwan NHIRD	Variable	Events 35 vs 3; PY 176,347 vs 5,602
**Ueda 2018 **[25]	SGLT2—i vs GLP-1 RA	VTE	HR	0.99	0.71	1.38	17,213 SGLT2—i users vs 17,213 GLP1-RA users	New-user, 1:1 PS-matched (active comparator)	Sweden & Denmark nationwide registers	9.0	Hospital ICD-10 outcomes; median follow-up ~ 270–274 days

### Risk of bias in studies

**Fig. 2:**
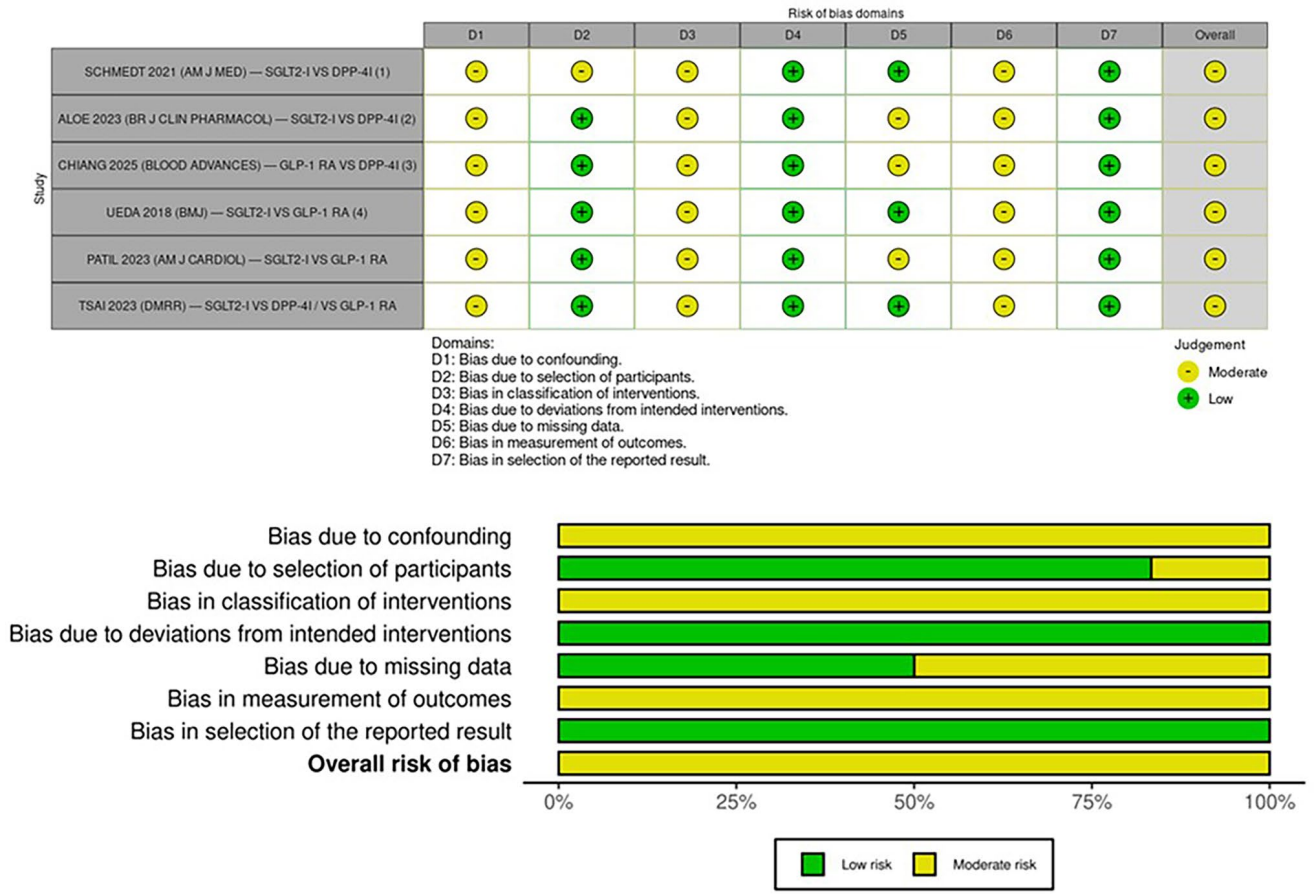
Traffic light plot of risk of bias and summary plot of risk of bias (ROBINS-I)

Across the 6 comparative studies, the overall risk of bias was consistently moderate (Fig. 2), with no study judged at serious risk [14, 21, 22, 24, 25]. Although all analyses applied robust adjustment methods (e.g., propensity score approaches or target-trial emulation), residual confounding remains plausible, particularly for factors such as obesity, smoking, and time-varying exposures. Most studies used new-user active-comparator designs, supporting internal validity, while one nested case–control study showed moderate concerns in participant selection. Outcome definitions relied on administrative codes and exposure classification on dispensing data, which may introduce misclassification. Detailed domain-level assessments are provided in the Supplementary Material. Randomized controlled trials were eligible; however, no studies meeting the predefined active-comparator criteria with extractable VTE outcomes were identified. Therefore, all included studies were non-randomized and assessed using ROBINS-I.

### Results of individual studies

Several comparisons included in this review are informed by single studies, particularly for GLP1-RA versus DPP-4i and for component outcomes (PE and DVT). These contrasts could not be meta-analyzed and are therefore reported descriptively. Accordingly, their estimates should be interpreted with caution, taking into account the absence of between-study synthesis and the potential for imprecision. Moreover, the small number of studies (*k* = 1–3 across comparisons) limits statistical power and precision, and precludes robust assessment of heterogeneity and publication bias.

A) SGLT2—i vs DPP-4i.

**Fig. 3:**
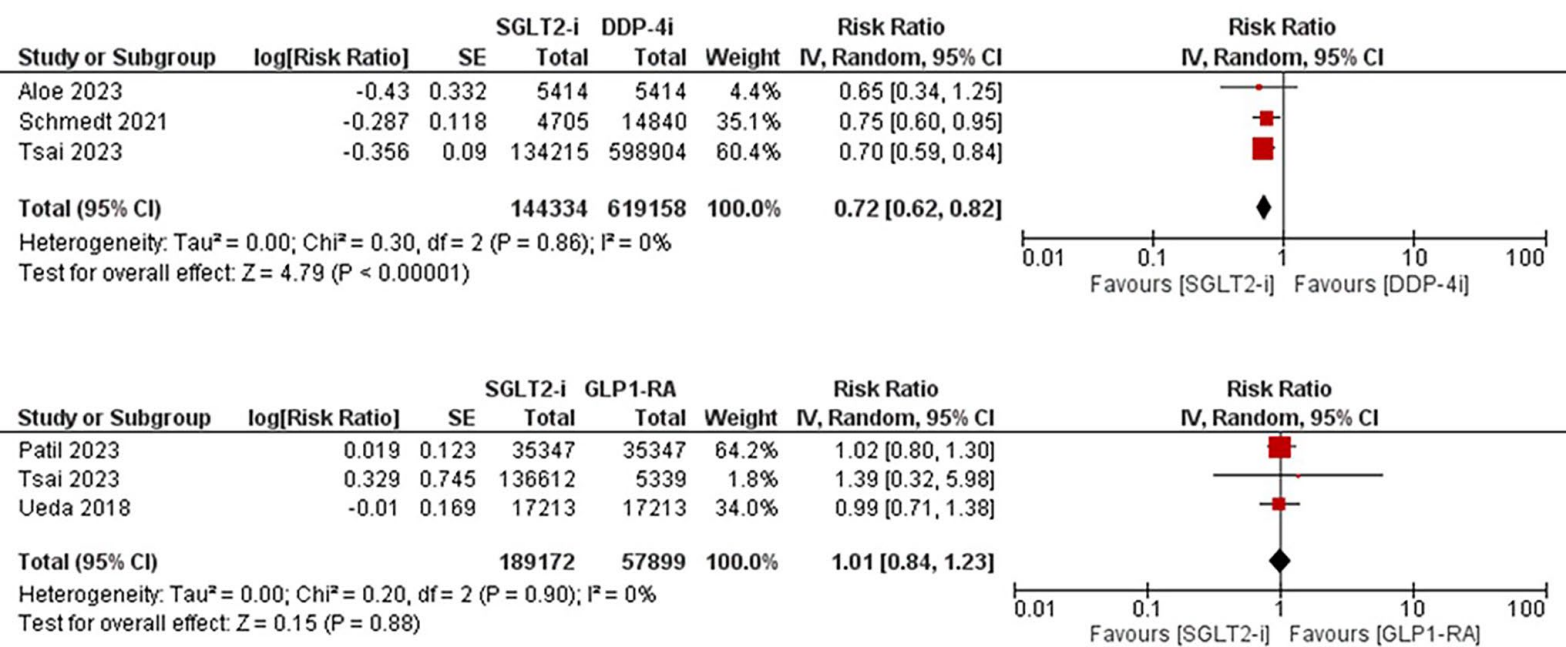
Forest plots of pooled analyses for risk of venous thromboembolism (VTE). In three studies comparing SGLT2 inhibitors with DPP-4 inhibitors, SGLT2 inhibitors were associated with a significantly lower risk (HR 0.72, 95% CI 0.62–0.82; I2 = 0%). In contrast, across three studies comparing SGLT2 inhibitors with GLP-1 receptor agonists, no significant difference in VTE risk was observed (HR 1.01, 95% CI 0.84–1.23; I2 = 0%)

About the primary outcome (VTE), three studies [21, 23, 24] yielded a pooled HR of 0.72 (95% CI 0.62–0.82) with low heterogeneity (Fig. 3). Results were consistent across fixed- and random-effects models and robust to study design differences. No heterogeneity was detected, although this estimate is unreliable given the small number of studies (I2 = 0%, τ2 = 0.00; *Q* = 0.30, *df* = 2, *p* = 0.86). PE and DVT components were reported only in the study by Tsai et al. (2023) [24]. Specifically, SGLT2 inhibitor use was associated with a lower risk of PE compared with DPP-4 inhibitors (HR 0.44, 95% CI 0.30–0.64), while a more modest reduction was observed for DVT (HR 0.82, 95% CI 0.68–0.99) [24].

B) GLP-1 RA vs DPP-4i.

About VTE outcome, one large target-trial emulation (Chiang 2025) showed HR 0.78 (0.73–0.83), favoring GLP-1 RA vs DPP-4i over 12 months [14] (Supplementary materials). Heterogeneity analysis is based on a single study (Chiang 2025; heterogeneity is not applicable *k* = 1). The pooled estimate equals the study estimate: HR 0.78 (95% CI 0.73–0.83). Between-study variance and I2 cannot be estimated, and a prediction interval is not computed. This comparison is based on a single large study and should be interpreted as hypothesis-generating rather than confirmatory. Regarding individual components, the same study showed a lower risk of PE (HR 0.74, 95% CI 0.68–0.82) and DVT (HR 0.81, 95% CI 0.75–0.88) with GLP1-RAs compared with DPP-4i [14].

C) SGLT2—i vs GLP-1 RA (head-to-head).

### Primary outcome (VTE)

Pooling Ueda 2018, Patil 2023, and the head-to-head arm of Tsai 2023 gave HR 1.02 (0.84–1.23), indicating no clear difference in VTE risk between classes [22, 24, 25] (Fig. 3). No heterogeneity was detected, although this estimate is unreliable given the small number of studies (I2 = 0%, τ2 = 0.00; *Q* = 0.20, *df* = 2, *p* = 0.90). The pooled random-effects estimate showed no difference between classes: RR 1.01 (95% CI 0.84–1.23), with the overall effect non-significant (*Z* = 0.15, *p* = 0.88). Data on individual components were available only from Tsai (2023) and were highly imprecise, with no clear differences observed for PE (HR 0.42, 95% CI 0.06–3.06) or DVT (HR 3.48, 95% CI 0.49–24.91) [24].

### Results of syntheses

Component-specific and subgroup analyses were based on single studies for several comparisons and are therefore reported for completeness and transparency only; these estimates should be considered descriptive and highly imprecise.

### Venous thromboembolism

Across three studies comparing SGLT2—i vs DPP-4i [21, 23, 24], the pooled estimate showed a 29% decrease in VTE risk with SGLT2—i (HR 0.72; 95% CI 0.62–0.82), consistent across heterogeneous designs. In the single large GLP1-RA vs DPP-4i target-trial emulation [14] (Chiang 2025; 270,129 per arm), GLP1-RAs were likewise associated with reduced VTE (HR 0.78; 95% CI 0.73–0.83). The head-to-head SGLT2—i vs GLP1-RA meta-analysis [22, 24, 25] indicated no clear difference, although estimates were imprecise (HR 1.02; 95% CI 0.84–1.23) (Table 2).

**Table 2:** Class-level comparisons of thromboembolic outcomes reported as pooled or single-study effect estimates with the number of contributing studies (k) and sources

Outcome	Comparison	K	Effect estimate [HR (95% CI)]	Study sources
**VTE**	SGLT2—i vs DPP-4I	3	0.72 (0.62–0.82)	Schmedt 2021 [23] Aloe 2023 [21] Tsai 2023 [24]
	GLP-1 RA vs DPP-4i	1	0.78 (0.73–0.83)	Chiang 2025 [14]
	SGLT2—i vs GLP1-RA	3	1.01 (0.84–1.23)	Ueda 2018 [25] Patil 2023 [22] Tsai 2023 [24]
**PE**	SGLT2—i vs DPP-4i	1	0.44 (0.30–0.64)	Tsai 2023 [24]
	GLP-1 RA vs DPP-4i	1	0.74 (0.68–0.82)	Chiang 2025 [14]
	SGLT2—i vs GLP-1 RA	1	0.42 (0.06–3.06)	Tsai 2023 [24]
**DVT**	SGLT2—i vs DPP-4i	1	0.82 (0.68–0.99)	Tsai 2023 [24]
	GLP-1 RA vs DPP-4i	1	0.81 (0.75–0.88)	Chiang 2025 [14]
	SGLT2—i vs GLP-1 RA	1	3.48 (0.49–24.91)	Tsai 2023 [24]

### Pulmonary embolism

Evidence by class comes from single studies. Versus DPP-4i, SGLT2—i were associated with substantially reduced PE risk [24] (HR 0.44; 95% CI 0.30–0.64; weighted pseudo-population 134,215 SGLT2—i vs 598,904 DPP-4i; 39 vs 439 events). GLP1-RA vs DPP-4i (Chiang 2025) [14] also favored GLP1-RAs (HR 0.74; 95% CI 0.68–0.82; 787 vs 1,024 PE events). The head-to-head estimate from Tsai [24] was imprecise (HR 0.42; 95% CI 0.06–3.06) due to very few GLP1-RA events (3 PE among 5,339 users) (Table 2).

### Deep vein thrombosis

For SGLT2—i vs DPP-4i, Tsai reported a modest reduction (HR 0.82; 95% CI 0.68–0.99; 200 vs 1,202 DVT events in the weighted cohorts) [24]. GLP1-RA vs DPP-4i (Chiang 2025) similarly showed decreased DVT risk with GLP1-RAs (HR 0.81; 95% CI 0.75–0.88; 1,063 vs 1,268 events) [14]. The SGLT2—i vs GLP1-RA head-to-head result from Tsai was very imprecise (HR 3.48; 95% CI 0.49–24.91) because only 2 DVT events occurred among 5,339 GLP1-RA users, yielding minimal weight in synthesis [24] (Table 2).

### Reporting biases

We explored small-study effects for comparisons with at least three contributing studies. For SGLT2—i vs DPP-4i (VTE; *k* = 3) and for the head-to-head SGLT2—i vs GLP1-RA (VTE; *k* = 3), we generated funnel plots of log effect versus standard error and performed Egger’s regression on the standard normal deviate (Supplementary materials). For SGLT2—i vs DPP-4i, the funnel plot appeared broadly symmetric, and Egger’s test did not suggest small-study effects (intercept − 4.15, SE 1.03; *t* =  − 4.05; df = 1; *p* = 0.154). For the head-to-head comparison, two studies with moderate precision clustered near the null, while one imprecise estimate (from a cohort with a very small GLP-1 RA arm) lay at high standard error; visual asymmetry was not evident and Egger’s test was likewise non-significant (intercept − 0.04, SE 0.16; *t* =  − 0.24; *df* = 1; *p* = 0.853). These diagnostics are underpowered with only three studies per comparison, and therefore do not allow reliable conclusions regarding the presence or absence of publication bias. We did not assess publication bias for PE or DVT because only a single study contributed to each class-wise contrast.

### Certainty of evidence

We applied GRADE to each comparison and outcome. Because all included studies are observational, certainty starts at Low and can be rated down for risk of bias, inconsistency, indirectness, imprecision, or publication bias, and up when appropriate. ROBINS-I was Moderate overall; publication-bias tests were underpowered (*k* = 3) (Table 3). To express absolute effects, we used 12-month event rates from the DPP-4i arm in Chiang 2025: VTE 7.6/1000, PE 3.8/1000, DVT 4.7/1000. Across ~ 12 months, both SGLT2 inhibitors and GLP-1 RAs reduce events versus DPP-4i with moderate certainty overall: for VTE, SGLT2—i vs DPP-4i HR 0.72 (0.62–0.82), 5.4 vs 7.6/1,000 (Δ − 2.2; − 2.9 to − 1.4); GLP-1 RA vs DPP-4i HR 0.78 (0.73–0.83), 5.9 vs 7.6/1,000 (Δ − 1.7; − 2.0 to − 1.3); SGLT2—i vs GLP-1 RA HR 1.02 (0.84–1.23), 7.8 vs 7.6/1,000 (Δ + 0.2; − 1.2 to + 1.8; low certainty). For PE, SGLT2—i vs DPP-4i HR 0.44 (0.30–0.64), 1.7 vs 3.8/1,000 (Δ − 2.1; − 2.7 to − 1.4; low–moderate certainty); GLP-1 RA vs DPP-4i HR 0.74 (0.68–0.82), 2.8 vs 3.8/1,000 (Δ − 1.0; − 1.2 to − 0.7; moderate); SGLT2—i vs GLP-1 RA HR 0.42 (0.06–3.06), 1.6 vs 3.8/1,000 (Δ − 2.2; − 3.6 to + 7.8; very low). For DVT, SGLT2—i vs DPP-4i HR 0.82 (0.68–0.99), 3.9 vs 4.7/1,000 (Δ − 0.85; − 1.50 to − 0.05; low–moderate); GLP-1 RA vs DPP-4i HR 0.81 (0.75–0.88), 3.8 vs 4.7/1,000 (Δ − 0.9; − 1.2 to − 0.6; moderate); SGLT2—i vs GLP-1 RA HR 3.48 (0.49–24.91), 16.4 vs 4.7/1,000 (Δ + 11.7; − 2.4 to + 112.4; very low). Overall, absolute differences are small but clinically relevant versus DPP-4i, while head-to-head estimates remain uncertain (especially for PE/DVT).

**Table 3:** Class-level comparisons are reported as HRs (95% CIs) and translated to absolute risks per 1000 patients over ~ 12 months

Outcome	Comparison	Relative effect (HR, 95% CI)	Assumed risk (PER 1000)	Corresponding risk (per 1000; 95% CI)	Absolute difference (per 1000; 95% CI)	Certainty (Grade)
VTE	SGLT2—i vs DPP-4i	0.72 (0.62–0.82)	7.59	5.39 (4.70–6.22)	− 2.20 (− 2.88 to − 1.37)	Moderate ⊕ ⊕ ⊕ ◯
VTE	GLP1-RA vs DPP-4i	0.78 (0.73–0.83)	7.59	5.92 (5.54–6.30)	− 1.67 (− 2.05 to − 1.29)	Moderate ⊕ ⊕ ⊕ ◯
VTE	SGLT2—i vs GLP1-RA	1.02 (0.84–1.23)	7.59	7.74 (6.37–9.33)	+ 0.15 (− 1.21 to + 1.74)	Low ⊕ ⊕ ◯◯
DVT	SGLT2—i vs DPP-4i	0.82 (0.68–0.99)	4.69	3.85 (3.19–4.65)	− 0.84 (− 1.50 to − 0.05)	Low–Moderate ⊕ ⊕ ◯◯
DVT	GLP1-RA vs DPP-4i	0.81 (0.75–0.88)	4.69	3.80 (3.52–4.13)	− 0.89 (− 1.17 to − 0.56)	Moderate ⊕ ⊕ ⊕ ◯
DVT	SGLT2—i vs GLP1-RA	3.48 (0.49–24.91)	4.69	16.34 (2.30–116.93)	+ 11.64 (-2.39 to + 112.23)	Very low ⊕ ◯◯◯
PE	SGLT2—i vs DPP-4i	0.44 (0.30–0.64)	3.79	1.67 (1.14–2.43)	− 2.12 (− 2.65 to − 1.36)	Low–Moderate ⊕ ⊕ ◯◯
PE	GLP1-RA vs DPP-4i	0.74 (0.68–0.82)	3.79	2.81 (2.58–3.11)	− 0.99 (− 1.21 to − 0.68)	Moderate ⊕ ⊕ ⊕ ◯
PE	SGLT2—i vs GLP1-RA	0.42 (0.06–3.06)	3.79	1.59 (0.23–11.60)	− 2.20 (− 3.56 to + 7.81)	Very low ⊕ ◯◯◯

Baseline risks (DPP-4i arm, 12-month follow-up from Chiang 2025): VTE 7.59/1000; PE 3.79/1000; DVT 4.69/1000. “Corresponding risk” = baseline × HR; “absolute difference” = corresponding risk − baseline (negative values = fewer events with the first-listed class). Certainty (GRADE): for VTE, Moderate for SGLT2—i or GLP-1 RA vs DPP-4i and Low for SGLT2—i vs GLP-1 RA (imprecision); for PE and DVT, SGLT2—i vs DPP-4i and GLP-1 RA vs DPP-4i are from single large studies, while SGLT2—i vs GLP-1 RA is very imprecise with sparse events, yielding very low certainty

### Sensitivity analyses: BMI adjustment and follow-up duration

**Fig. 4:**
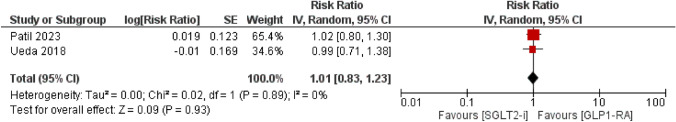
Forest plots for VTE (BMI-adjusted only). Random-effects pooled estimates are restricted to studies adjusting for BMI/adiposity in primary models

When restricting to studies that adjusted for BMI/adiposity, findings were consistent with the main analyses. For GLP1-RAs versus DPP-4i, the pooled HR for VTE remained 0.78 (95% CI 0.73–0.83; *k* = 1) [14]. For SGLT2—i versus DPP-4i, the protective signal persisted but with wider confidence intervals, as only Aloe et al. contributed (HR 0.65, 95% CI 0.34–1.25) [21]. For SGLT2—i versus GLP1-RAs, the pooled estimate across two BMI-adjusted cohorts was neutral (HR 1.01, 95% CI 0.83–1.23; *k* = 2) [22, 25] (Fig. 4).

When stratified by follow-up duration, studies with ≤ 12 months of follow-up showed pooled estimates consistent with the main analysis across all comparisons. Estimates from > 12 months/variable follow-up cohorts were broadly similar but less precise and potentially influenced by asymmetric exposure time [22]. In meta-regression, follow-up duration was not significantly associated with effect size for any comparison (slope per + 12 months: SGLT2i vs DPP-4i − 2% [95% CI − 14 to + 12]; SGLT2i vs GLP-1RA + 4% [− 8 to + 17]; both *p* > 0.10) (Supplementary materials).

## Discussion

Across 6 comparative studies [14, 21–25] spanning multiple regions and data sources, two consistent signals emerge. First, both SGLT2—i and GLP1-RAs showed a lower incidence of VTE risk than DPP-4i [14, 21–25]. This pattern appears in 3 heterogeneous SGLT2—i vs DPP-4i studies and in one large TTE of GLP1-RA vs DPP-4i, suggesting the finding is not driven by any single design [14, 21, 23, 24]. Second, the head-to-head comparison (SGLT2—i vs GLP1-RA) shows no material difference in VTE risk (pooled HR 1.02), with the most imprecise estimate contributed by the small GLP1-RA cohort in Tsai and therefore carrying minimal meta-analytic weight [22, 24, 25]. Although VTE is a relatively infrequent outcome, this is consistent with both randomized and real-world evidence. Low absolute incidence does not preclude clinical relevance, particularly when differences are evaluated across large treated populations and in the context of severe outcomes such as PE. To address this issue, we translated relative estimates into absolute risks over approximately 12 months and assessed certainty using the GRADE framework. This approach shows that absolute risk reductions versus DPP-4i are modest but potentially meaningful at the population level, while head-to-head comparisons between SGLT2—i and GLP1-RA remain uncertain. Notably, the limited number of studies and the reliance on few or single-study estimates restrict statistical power, increase uncertainty, and prevent robust assessment of heterogeneity and publication bias.

Our class-to-class results fit with randomized evidence showing no excess VTE risk with SGLT2—i in trials (meta-analysis RR 0.98), arguing against drug-induced prothrombotic effects despite hemoconcentration concerns [26]. Indeed, in the work of Lewis et al., among ~ 269k matched patients, SGLT2—i initiation increased erythrocytosis prevalence, but erythrocytosis was not associated with higher venous or arterial thrombosis; hemoglobin/hematocrit rose modestly [26].

Otherwise, the meta-analysis by Liu et al. demonstrated a significant rise in DVT risk with GLP1-RAs versus placebo or non-GLP1-RAs, while not detecting a significant increase in overall VTE; this effect was more pronounced in trials of longer duration (> 18 months) and in CVOTs [20]. By contrast, our work, which focuses on active comparators (GLP1-RAs or SGLT2—i vs DPP-4i, and head-to-head), demonstrated a reduction in VTE risk for both GLP1-RAs and SGLT2—i compared with DPP-4i, and no significant difference between the two newer drug classes, although estimates were imprecise. Several features of the evidence base help reconcile these differences. First, the choice of comparator matters: placebo-controlled RCTs estimate how GLP1-RAs perform against “no active therapy of that class,” whereas our analyses reflect the real clinical choice between drug classes. DPP-4—i have generally been VTE-neutral in CVOTs and observational syntheses, reinforcing the value of active-comparator designs to minimize channeling and confounding [21, 22, 25]. Second, the time on treatment is not uniform across studies. The DVT signal highlighted in the meta-analysis by Liu is largely driven by long-duration CVOTs, while many real-world cohorts observe patients for about one year, a window in which such signals may not fully emerge [20]. Our analysis by duration of follow-up indicated that the observed class effects within approximately one year persist over longer horizons, without evidence of systematic attenuation or reversal. However, these findings should be interpreted cautiously, given heterogeneity in study design, asymmetric follow-up across treatment arms in some cohorts, and the ecological nature of study-level meta-regression. Overall, we found no signal that longer follow-up modifies the comparative risk of VTE, but dedicated long-term head-to-head studies remain needed.

DVT is a relatively rare endpoint in RCTs, so results can be sensitive to how zero-event trials are handled and to modeling choices (e.g., fixed- vs random-effects), potentially nudging estimates in either direction. Finally, the individual components of VTE may not follow the same pattern. For instance, Chiang et al. observed a reduction in PE with GLP1-RAs, suggesting that the risks of DVT and PE may diverge depending on clinical setting and length of follow-up [19].

At the disease level, whether type 2 diabetes independently increases VTE risk remains debated, as associations often attenuate after adjustment for adiposity and related factors, suggesting residual confounding in observational data [1, 27–30]. Our findings—lower VTE risk versus DPP-4i and neutrality in head-to-head comparisons—are consistent with this perspective and with the hypothesis of a prothrombotic milieu in diabetes that does not consistently translate into large clinical effects [8, 14]. Supporting evidence from specific clinical settings is limited but directionally consistent. For example, in a national claims analysis of patients undergoing total knee arthroplasty, preoperative semaglutide exposure was associated with a lower risk of adverse events, including VTE [10].

From a methodological perspective, the included studies provide complementary evidence across different designs. Large target-trial emulations offer precise estimates, while cohort and case–control studies reinforce consistency across settings [14, 21, 23]. In head-to-head comparisons, imprecision—particularly in studies with small GLP-1 RA samples—likely reflects limited events rather than systematic bias [22, 24, 25].

We provide direct active-comparator and head-to-head estimates between drug classes commonly used in clinical practice. Both GLP1-RAs and SGLT2—i were associated with lower VTE risk compared with DPP-4i, with no meaningful difference between the two classes. Translating these findings into absolute 12-month risks and GRADE assessment indicates moderate certainty for comparisons versus DPP-4i and lower certainty for head-to-head estimates due to sparse events. These results are consistent with trial-based signals of reduced PE with GLP-1RAs and with large real-world studies showing a VTE benefit versus DPP-4i, while helping contextualize the DVT signal reported in recent placebo-based meta-analyses [19, 20]. Overall, the lower VTE, PE, and DVT risk observed versus DPP-4i provides a reassuring safety signal for modern glucose-lowering agents [14, 21, 23, 24]. Although absolute risk reductions are small at the individual level, they may be clinically relevant at the population level, particularly given the severity of outcomes such as pulmonary embolism. These findings are biologically plausible, as both GLP1-RAs and SGLT2—i exert pleiotropic effects beyond glycemic control, including improvements in endothelial function and reductions in systemic inflammation, which may counterbalance the prothrombotic milieu associated with type 2 diabetes. The absence of a clear difference in VTE risk between SGLT2—is and GLP1-RA in head-to-head comparisons likely reflects a balance of distinct but converging mechanisms. Although SGLT2—is are associated with hemoconcentration, this effect appears to be offset by improvements in inflammation and endothelial function. Similarly, GLP1-RAs promote weight loss and anti-inflammatory effects without clear additional antithrombotic benefit. Overall, the observed neutrality is biologically coherent and suggests that neither class exerts a clinically meaningful prothrombotic effect.

For clinicians choosing between SGLT2 inhibitors and GLP1-RAs, our findings indicate no clinically meaningful difference in VTE risk. Given the small absolute event rates, VTE considerations are unlikely to be a primary driver of treatment selection, but may provide reassurance regarding safety in patients at elevated thrombotic risk. Treatment decisions can therefore prioritize cardiorenal benefits, weight reduction, glycemic control, tolerability, and patient preferences rather than VTE risk [14, 22, 24, 25]. Evidence comparing these agents with metformin as an active comparator remains limited. Observational data suggest a lower VTE risk with metformin versus sulfonylureas, but direct comparisons with GLP1-RAs or SGLT2 inhibitors are lacking [31]. Ongoing trials (e.g., SMARTEST) may inform first-line decisions, but VTE endpoints remain underreported [32]. Sensitivity analyses restricted to BMI-adjusted studies confirmed the robustness of our findings, suggesting that residual confounding by adiposity is unlikely to fully explain the observed associations [14, 21, 22, 25]. Future research should focus on long-term head-to-head comparisons with standardized VTE definitions and improved adjustment for key confounders.

## Limitations of the study

The primary limitation of this study is the small number of eligible active-comparator studies, with only three studies contributing to the primary VTE analysis and several secondary analyses relying on single studies. This limits statistical power, precision, and the ability to explore heterogeneity, and precludes definitive conclusions. Our conclusions are largely on observational evidence, where even careful adjustment cannot fully eliminate residual confounding; accordingly, most studies were judged to have a moderate risk of bias. The generalizability beyond 12 months remains uncertain, given that the DVT signal in the work of Liu emerged primarily in trials exceeding 1.5 years [20]. Residual confounding and channeling bias remain important concerns, particularly given potential differences in baseline risk profiles and incomplete adjustment for key VTE risk factors such as malignancy or thrombophilia.

## Conclusions

In adults with diabetes, SGLT2—i and GLP1-RA are associated with a lower risk of VTE compared with DPP-4i, while head-to-head differences between the two classes appear neutral [8, 14, 22, 24, 25]. Those results add to a landscape where RCT meta-analyses have shown discordant signals across VTE components (PE vs DVT) and depending on the comparator [19, 20]; our synthesis, based on active comparators, provides estimates directly relevant to therapeutic choices. These findings should be interpreted with caution given the limited evidence base, imprecision of several estimates, and the observational nature of the included studies. In clinical practice, these findings suggest that VTE risk should not be a primary determinant of treatment selection between SGLT2—i and GLP1-RAs. Instead, therapeutic decisions should be guided by cardiorenal benefits, effects on body weight, glycemic needs, tolerability, and individual patient characteristics and preferences.

## Supplementary Information

Below is the link to the electronic supplementary material.

Supplementary file1 (DOCX 400 KB)

Supplementary file2 (DOCX 270 KB)

Supplementary file3 (DOCX 28 KB)

## Data Availability

Data are available upon request from the authors.
