# Supplementary material for: Venous thromboembolism with modern glucose-lowering agents in diabetes: active-comparator evidence beyond placebo-based meta-analyses
Source: Intern Emerg Med. 2026 May 6;21(4):1455–68. doi: 10.1007/s11739-026-04363-5 (PMC13263266; doi:10.1007/s11739-026-04363-5)
Supplement: Supplementary file 1 — Supplementary file1 (DOCX 400 KB) [file 11739_2026_4363_MOESM1_ESM.docx]

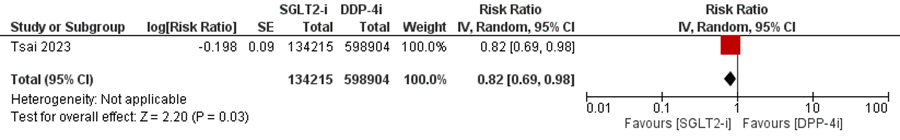


*Figure 1 Supplemental. Risk of deep venous thrombosis (DVT) with SGLT2 inhibitors versus DPP-4 inhibitors. Hazard ratios/risk ratios (HR/RR with 95% CI). Values <1 suggest a lower risk of VTE with SGLT2-i compared to DPP-4i.*


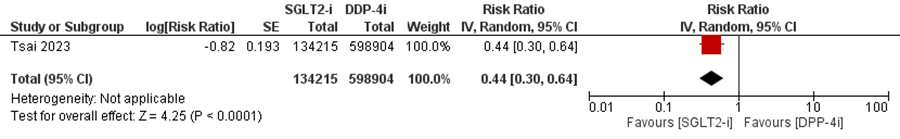


*Figure 2 Supplemental. Risk of pulmonary embolism (PE) with SGLT2 inhibitors versus DPP-4 inhibitors. Hazard ratios (HR with 95% CI). Values <1 suggest a lower risk of VTE with SGLT2-i compared to DPP-4i.*


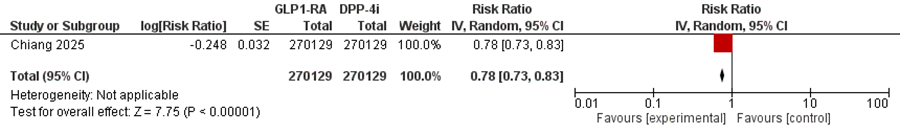


*Figure 3 Supplemental. Risk of venous thromboembolism (VTE) with GLP-1 receptor agonists versus DPP-4 inhibitors. Hazard ratio (HR with 95% CI) from Chiang 2025 (Blood Advances). Values <1 indicate a lower risk of VTE with GLP1-RAs compared to DPP-4i.*


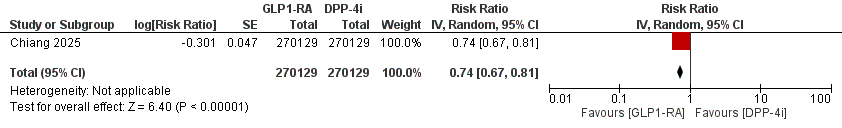


*Figure 4 Supplemental. Risk of pulmonary embolism (PE) with GLP-1 receptor agonists versus DPP-4 inhibitors. Hazard ratio (HR with 95% CI) from Chiang 2025 (Blood Advances). Values <1 indicate a lower risk of PE with GLP-1 receptor agonists compared to DPP-4 inhibitors.*


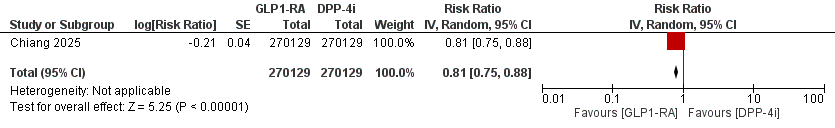


*Figure 5 Supplemental. Risk of deep vein thrombosis (DVT) with GLP-1 receptor agonists versus DPP-4 inhibitors. Hazard ratio (HR with 95% CI) from Chiang 2025 (Blood Advances). Values <1 indicate a lower risk of DVT with GLP-1 receptor agonists compared to DPP-4 inhibitors.*


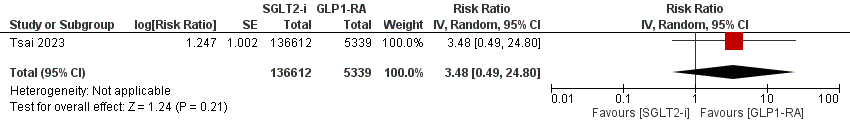


*Figure 6 Supplemental. Risk of deep venous thrombosis (DVT) with SGLT2 inhibitors versus GLP-1 receptor agonists. Values <1 indicate a lower risk of VTE with SGLT2 inhibitors compared to GLP-1 receptor agonists.*


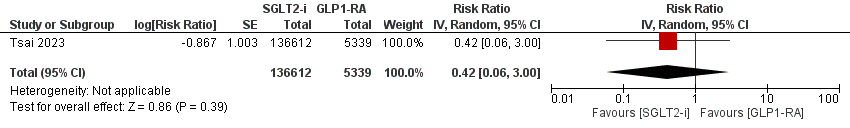


*Figure 7 Supplemental. Risk of pulmonary embolism (PE) with SGLT2 inhibitors versus GLP-1 receptor agonists. Values <1 indicate a lower risk of VTE with SGLT2 inhibitors compared to GLP-1 receptor agonists.*


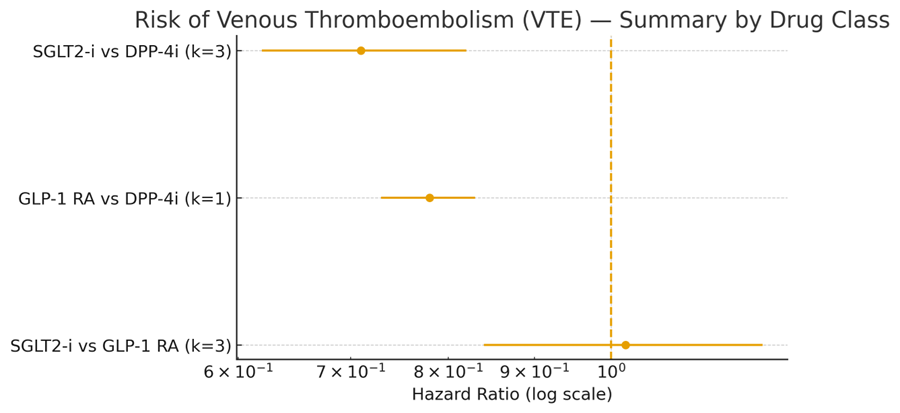


*Figure 8 Supplemental. Risk of Venous Thromboembolism (VTE) — Summary by Drug Class. Forest plot of pooled hazard ratios (HRs) comparing SGLT2 inhibitors vs DPP-4 inhibitors (k=3; Schmedt 2021, Aloe 2023, Tsai 2023), GLP-1 receptor agonists vs DPP-4 inhibitors (k=1; Chiang 2025), and SGLT2 inhibitors vs GLP-1 receptor agonists (k=3; Ueda 2018, Patil 2023, Tsai 2023). Estimates are shown on a log scale with 95% CIs; values <1 favor the first-listed class. Pooled results use inverse-variance fixed effects for summaries.*


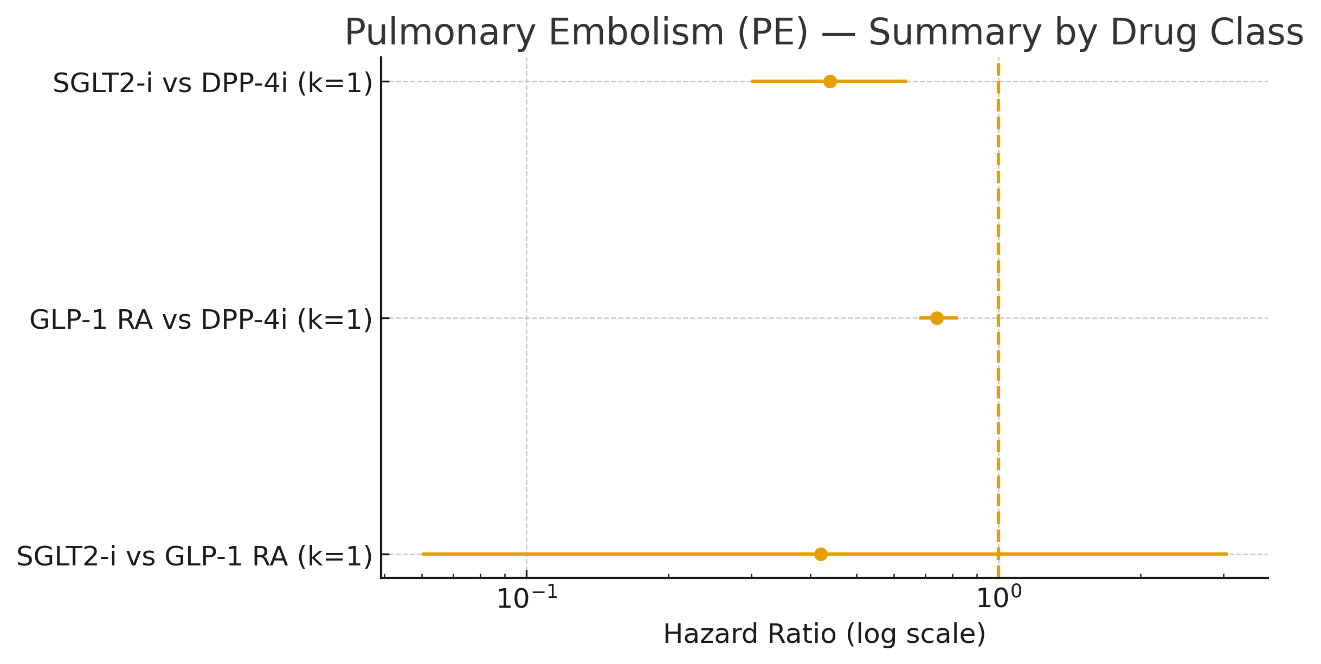


*Figure 9 Supplemental. Pulmonary Embolism (PE) — Summary by Drug Class. Class-level hazard ratios (HRs) for PE: SGLT2 inhibitors vs DPP-4 inhibitors (k=1; Tsai 2023), GLP-1 receptor agonists vs DPP-4 inhibitors (k=1; Chiang 2025), and SGLT2 inhibitors vs GLP-1 receptor agonists (k=1; Tsai 2023). Points represent study estimates with 95% CIs on a log scale; <1 favors the first-listed class.*


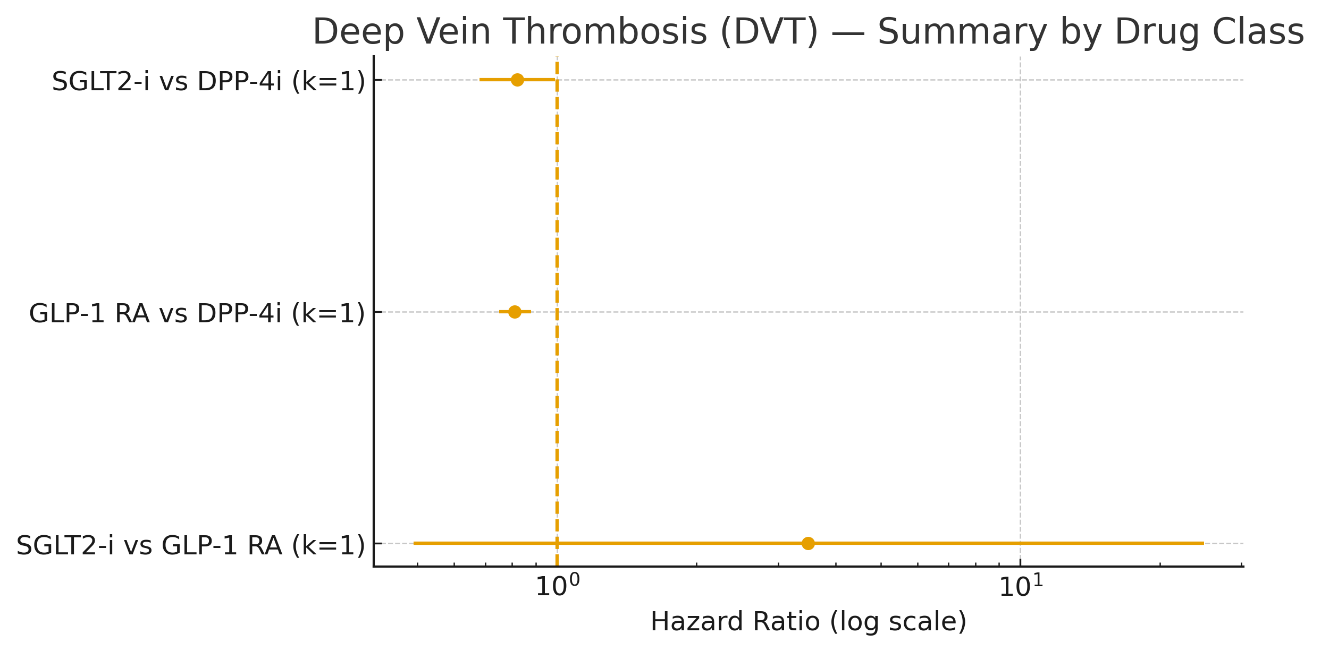


*Figure 10 Supplemental. Deep Vein Thrombosis (DVT) — Summary by Drug Class. Class-level hazard ratios (HRs) for DVT: SGLT2 inhibitors vs DPP-4 inhibitors (k=1; Tsai 2023), GLP-1 receptor agonists vs DPP-4 inhibitors (k=1; Chiang 2025), and SGLT2 inhibitors vs GLP-1 receptor agonists (k=1; Tsai 2023). Estimates shown on a log scale with 95% CIs; <1 favors the first-listed class. The head-to-head estimate is imprecise owing to sparse events in the GLP-1 RA arm.*


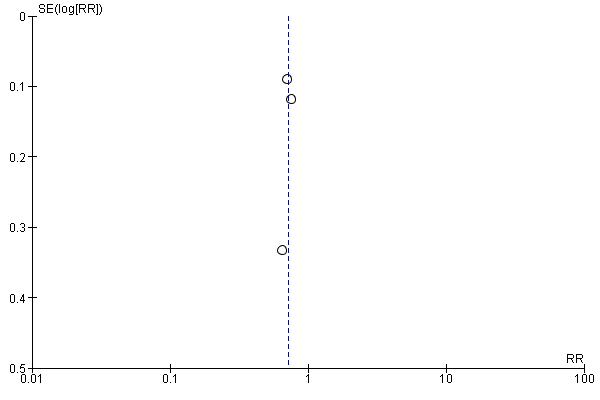


*Figure 11 Supplemental. Funnel plot of log(HR) versus SE for SGLT2-i vs DPP-4i (VTE). Dashed line shows fixed-effect pooled log(HR). With k=3, the plot is symmetric by inspection; Egger’s test does not indicate small-study effects, acknowledging limited power.*


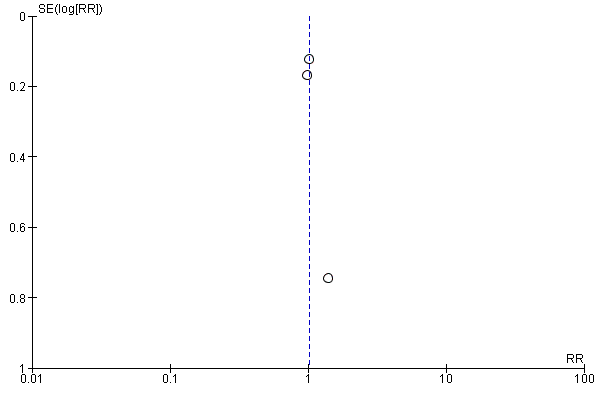


*Figure 12 Supplemental. Funnel plot for head-to-head SGLT2-i vs GLP1-RAs agonists VTE. Scatter of study precision (SE[log RR]) against effect size (RR, log scale). The dashed vertical line marks the pooled fixed-effect estimate (≈1.0). Two studies with moderate precision (Ueda 2018; Patil 2023) cluster near the null, while one imprecise study (Tsai 2023; small GLP-1 RA arm) lies lower in the plot with a wide SE. Visual inspection shows no clear asymmetry; however, with k=3 the funnel plot and any small-study tests are underpowered and should be interpreted cautiously.*


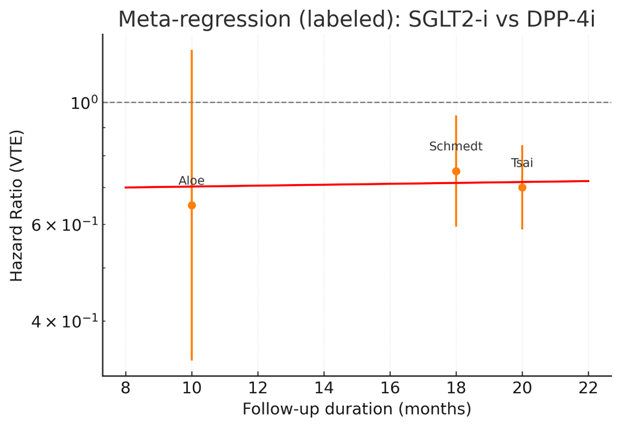


*Figure 13 Supplemental. Meta-regression of follow-up duration: SGLT2-i vs DPP-4i. Study-level HRs for VTE (95% CI) plotted against follow-up duration (months); labels indicate first author. The red line depicts the fitted meta-regression on log(HR); the grey dashed line indicates HR=1. Y-axis is log scale.*


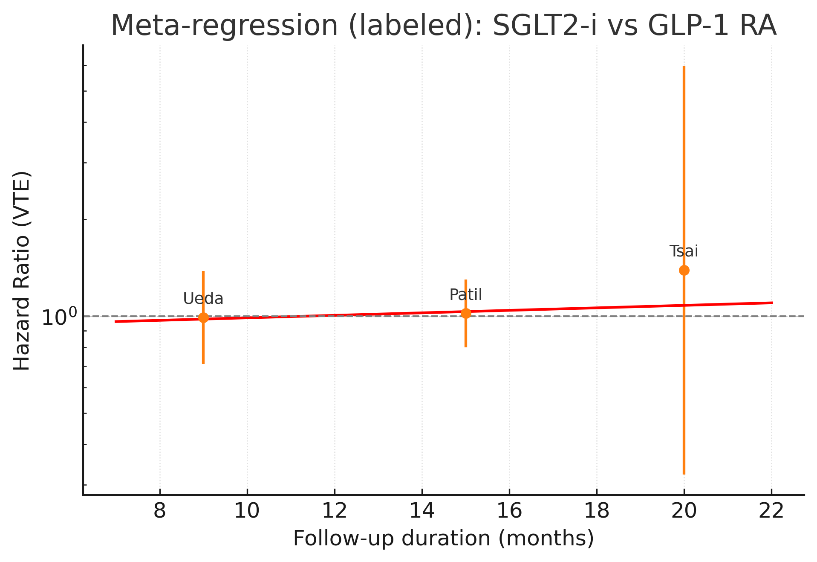


*Figure 14 Supplemental. Meta-regression of follow-up duration: SGLT2-i vs GLP1-RA. Each point is a study-level HR for VTE (95% CI) positioned by mean/median follow-up (months); labels denote first author. The red line is the weighted regression of log(HR) on follow-up duration; the dashed line marks HR=1. Y-axis is log scale.*
