# Supplementary material for: Venous thromboembolism with modern glucose-lowering agents in diabetes: active-comparator evidence beyond placebo-based meta-analyses
Source: Intern Emerg Med. 2026 May 6;21(4):1455–68. doi: 10.1007/s11739-026-04363-5 (PMC13263266; doi:10.1007/s11739-026-04363-5)
Supplement: Supplementary file 3 — Supplementary file3 (DOCX 28 KB) [file 11739_2026_4363_MOESM3_ESM.docx]

# Search Strategy

## Objective

To systematically assess and quantify the risk of venous thromboembolism (VTE) associated with GLP-1 receptor agonists (GLP1-RAs) and SGLT2 inhibitors, versus active comparators (DPP-4 inhibitors) and head-to-head (SGLT2-i vs GLP1-RA), also considering pulmonary embolism (PE) and deep vein thrombosis (DVT) separately.

## Eligibility (for screening)

**Population:** Adults (≥18 years).

**Designs:** Comparative observational (new-user cohorts, target trial emulations, nested case–control) and randomized trials reporting VTE/PE/DVT.

**Comparators:** DPP-4 inhibitors or head-to-head SGLT2i vs GLP1-RA.

**Outcomes:** VTE (composite) and/or PE, DVT.

**Limits:** Humans; no language restrictions.

**Time window:** Database inception to 16 Sep 2025.

**Exclusions:** Case reports/series, non-comparative designs, pediatric-only, in vitro/animal, narrative reviews.

## Information sources

MEDLINE/PubMed, Embase, Web of Science Core Collection, Scopus, Cochrane CENTRAL.

Grey literature: medRxiv and Research Square (title/abstract screening).

## PubMed (MEDLINE) search string

(
 ("Glucagon-Like Peptide 1 Receptor Agonists"[Mesh] OR "glucagon-like peptide-1 receptor agonist*"[tiab] OR GLP-1[tiab] OR "GLP-1 RA*"[tiab] OR liraglutide[tiab] OR semaglutide[tiab] OR dulaglutide[tiab] OR exenatide[tiab] OR lixisenatide[tiab])
 OR
 ("Sodium-Glucose Transporter 2 Inhibitors"[Mesh] OR "sodium-glucose co-transporter 2"[tiab] OR SGLT2[tiab] OR "SGLT-2"[tiab] OR canagliflozin[tiab] OR dapagliflozin[tiab] OR empagliflozin[tiab] OR ertugliflozin[tiab] OR ipragliflozin[tiab])
)
AND
(
 ("Dipeptidyl-Peptidase IV Inhibitors"[Mesh] OR "DPP-4 inhibitor*"[tiab] OR sitagliptin[tiab] OR saxagliptin[tiab] OR linagliptin[tiab] OR alogliptin[tiab] OR vildagliptin[tiab]
 OR
 ("Glucagon-Like Peptide 1 Receptor Agonists"[Mesh] AND "Sodium-Glucose Transporter 2 Inhibitors"[Mesh])
)
AND
(
 "Venous Thromboembolism"[Mesh] OR "Venous Thrombosis"[Mesh] OR "Pulmonary Embolism"[Mesh]
 OR (venous[tiab] AND (thromboembol*[tiab] OR thrombosis[tiab]))
 OR VTE[tiab] OR PE[tiab] OR DVT[tiab] OR "pulmonary embol*"[tiab] OR "deep vein thromb*"[tiab]
)
NOT (animals[mh] NOT humans[mh])

## Embase (Elsevier) search string

(
 'glucagon like peptide 1 receptor agonist'/exp OR 'glucagon like peptide 1 receptor agonist*':ti,ab OR glp-1:ti,ab OR liraglutide:ti,ab OR semaglutide:ti,ab OR dulaglutide:ti,ab OR exenatide:ti,ab OR lixisenatide:ti,ab
 OR
 'sodium glucose cotransporter 2 inhibitor'/exp OR 'sodium glucose co-transporter 2':ti,ab OR sglt2:ti,ab OR canagliflozin:ti,ab OR dapagliflozin:ti,ab OR empagliflozin:ti,ab OR ertugliflozin:ti,ab OR ipragliflozin:ti,ab
)
AND
(
 'dipeptidyl peptidase 4 inhibitor'/exp OR 'dpp-4 inhibitor*':ti,ab OR sitagliptin:ti,ab OR linagliptin:ti,ab OR saxagliptin:ti,ab OR alogliptin:ti,ab OR vildagliptin:ti,ab
 OR
 ('glucagon like peptide 1 receptor agonist'/exp AND 'sodium glucose cotransporter 2 inhibitor'/exp)
)
AND
(
 'venous thromboembolism'/exp OR 'venous thrombosis'/exp OR 'pulmonary embolism'/exp
 OR (venous:ti,ab NEAR/3 (thromboembol*:ti,ab OR thrombosis:ti,ab))
 OR vte:ti,ab OR pe:ti,ab OR dvt:ti,ab OR 'pulmonary embol*':ti,ab OR 'deep vein thromb*':ti,ab
)
AND [humans]/lim AND [adult]/lim

## Web of Science Core Collection search (Topic)

TS=(
 (("glucagon-like peptide-1" NEAR/3 (agonist OR agonists OR receptor*)) OR GLP-1 OR liraglutide OR semaglutide OR dulaglutide OR exenatide OR lixisenatide
 OR ("sodium-glucose" NEAR/3 (cotransporter OR co-transporter)) OR SGLT2 OR canagliflozin OR dapagliflozin OR empagliflozin OR ertugliflozin OR ipragliflozin)
 AND
 ((DPP-4 NEAR/1 inhibitor*) OR sitagliptin OR saxagliptin OR linagliptin OR alogliptin OR vildagliptin
 OR ((GLP-1 OR liraglutide OR semaglutide OR dulaglutide OR exenatide OR lixisenatide) AND (SGLT2 OR canagliflozin OR dapagliflozin OR empagliflozin OR ertugliflozin OR ipragliflozin)))
 AND
 ((venous NEAR/3 (thromboembol* OR thrombosis)) OR "venous thromboembolism" OR "deep vein thromb*" OR "pulmonary embol*"
 OR VTE OR DVT OR PE)
)
Timespan: 1900–2025-09-16; Indexes: SCI-EXPANDED, SSCI, ESCI

## Scopus search (TITLE-ABS-KEY)

TITLE-ABS-KEY(
 (
 ("glucagon-like peptide-1" W/3 (agonist OR agonists OR receptor*)) OR GLP-1 OR liraglutide OR semaglutide OR dulaglutide OR exenatide OR lixisenatide
 OR ("sodium-glucose" W/3 (cotransporter OR co-transporter)) OR SGLT2 OR canagliflozin OR dapagliflozin OR empagliflozin OR ertugliflozin OR ipragliflozin
 )
 AND
 (
 ("DPP-4" W/1 inhibitor*) OR sitagliptin OR saxagliptin OR linagliptin OR alogliptin OR vildagliptin
 OR ( (GLP-1 OR liraglutide OR semaglutide OR dulaglutide OR exenatide OR lixisenatide) AND (SGLT2 OR canagliflozin OR dapagliflozin OR empagliflozin OR ertugliflozin OR ipragliflozin) )
 )
 AND
 (
 ("venous thromboembolism") OR ("deep vein thromb*") OR ("pulmonary embol*")
 OR (venous W/3 (thromboembol* OR thrombosis)) OR VTE OR DVT OR PE
 )
)

## Cochrane CENTRAL search

([mh "Glucagon-Like Peptide 1 Receptor Agonists"] OR "glucagon-like peptide-1" NEXT agonist* OR liraglutide OR semaglutide OR dulaglutide OR exenatide OR lixisenatide
 OR [mh "Sodium-Glucose Transporter 2 Inhibitors"] OR SGLT2 OR canagliflozin OR dapagliflozin OR empagliflozin OR ertugliflozin OR ipragliflozin)
AND
([mh "Dipeptidyl-Peptidase IV Inhibitors"] OR "DPP-4 inhibitor*" OR sitagliptin OR saxagliptin OR linagliptin OR alogliptin OR vildagliptin
 OR ([mh "Glucagon-Like Peptide 1 Receptor Agonists"] AND [mh "Sodium-Glucose Transporter 2 Inhibitors"]))
AND
([mh "Venous Thromboembolism"] OR [mh "Venous Thrombosis"] OR [mh "Pulmonary Embolism"] OR "venous thromboembolism" OR "pulmonary embol*" OR "deep vein thromb*")

## Grey literature (medRxiv, Research Square)

("glucagon-like peptide-1" OR GLP-1 OR liraglutide OR semaglutide OR dulaglutide OR exenatide OR lixisenatide
 OR "sodium-glucose" OR SGLT2 OR canagliflozin OR dapagliflozin OR empagliflozin OR ertugliflozin OR ipragliflozin)
AND
("venous thromboembolism" OR VTE OR "pulmonary embol*" OR PE OR "deep vein thromb*" OR DVT)
